# Immune Response to Mucosal *Brucella* Infection

**DOI:** 10.3389/fimmu.2019.01759

**Published:** 2019-08-20

**Authors:** Rubén López-Santiago, Ana Beatriz Sánchez-Argáez, Liliana Gabriela De Alba-Núñez, Shantal Lizbeth Baltierra-Uribe, Martha Cecilia Moreno-Lafont

**Affiliations:** ^1^Departamento de Inmunología, Escuela Nacional de Ciencias Biológicas, Instituto Politécnico Nacional, Mexico City, Mexico; ^2^Departamento de Microbiología, Escuela Nacional de Ciencias Biológicas, Instituto Politécnico Nacional, Mexico City, Mexico

**Keywords:** brucellosis, gut immunity, *Brucella* vaccines, intracellular infection, respiratory tract immunity

## Abstract

Brucellosis is one of the most prevalent bacterial zoonosis of worldwide distribution. The disease is caused by *Brucella* spp., facultative intracellular pathogens. Brucellosis in animals results in abortion of fetuses, while in humans, it frequently manifests flu-like symptoms and a typical undulant fever, being osteoarthritis a common complication of the chronic infection. The two most common ways to acquire the infection in humans are through the ingestion of contaminated dairy products or by inhalation of contaminated aerosols. *Brucella* spp. enter the body mainly through the gastrointestinal and respiratory mucosa; however, most studies of immune response to *Brucella* spp. are performed analyzing models of systemic immunity. It is necessary to better understand the mucosal immune response induced by *Brucella* infection since this is the main entry site for the bacterium. In this review, some virulence factors and the mechanisms needed for pathogen invasion and persistence are discussed. Furthermore, some aspects of local immune responses induced during *Brucella* infection will be reviewed. With this knowledge, better vaccines can be designed focused on inducing protective mucosal immune response.

## Introduction

The discovery of brucellosis took place in Malta during the Crimean war in 1859. The British troops had been suffering high fever, so the Royal British medical staff was called in. David Bruce was the officer who investigated what he called Malta fever or Mediterranean fever. In 1887, he managed to isolate and cultivate the bacterium responsible for the disease (1).

Later, Themistocles Zammit found that people who lived in farms, reared goats, and drank milk from these animals showed the same symptoms as Mediterranean fever patients. Using this finding, Zammit reproduced the infection in healthy goats and successfully isolated the bacterium in blood and milk. He deduced that the British army contracted the infection by consuming milk from infected animals in the local region. Therefore, in 1906 a decision was made to ban goat's milk consumption as a preemptive measure to control the disease among the British army. However, Malta fever was not eradicated in the region and suspicions arose regarding the consumption of ice-cream, cheese, and fudge made from contaminated milk (1, 2).

Zammit's contribution proved that brucellosis is mostly transmitted orally. Later, other contagion routes were reported (respiratory, parenteral, or by contact) and considered occupational hazards.

Although it has been well-established that *Brucella* enters the organism orally, the bacterium has not been properly defined as enteropathogenic, which has caused certain controversy. The infection by *Brucella* spp. does not cause diarrhea, a characteristic symptom of enteropathogenic bacteria (3, 4). Diarrhea is caused by inflammation due to the recruitment of cells, as neutrophils, producing damage to the epithelium, and compromising the integrity of the mucosa, which triggers inflammatory diarrhea (5). Studies *in vivo* have demonstrated that mice intragastrically inoculated with *B. melitensis* do not recruit neutrophils in the small intestine, while histological sections do not show considerable inflammation (6). On the contrary, enteropathogens as *Salmonella, Vibrio cholera*, and *Escherichia coli* do cause the characteristic inflammatory reaction and trigger diarrhea (5). Additionally, *Brucella* seems to transit only the intestine and does not appear to invade it and create as replicative niche in it (3, 4).

Animal models in which *Brucella* spp. is orally or intragastrically inoculated show that the bacterium can be recovered from the small intestine, particularly from gut-associated lymphoid tissue (GALT) as Peyer's patches (PP) and mesenteric lymph nodes (MLN). The bacterium is located in these tissues from an early stage of the infection and up to 21 days afterwards, as demonstrated by plating homogenates from the organs. This suggests that *Brucella* remains at these sites to replicate (6). However, infection in these models is achieved using high bacterial doses, 10^8^-10^10^ colony forming units (CFU), which might force gastrointestinal tract tissues to be colonized by the bacterium (3, 4).

**Figure 1:**
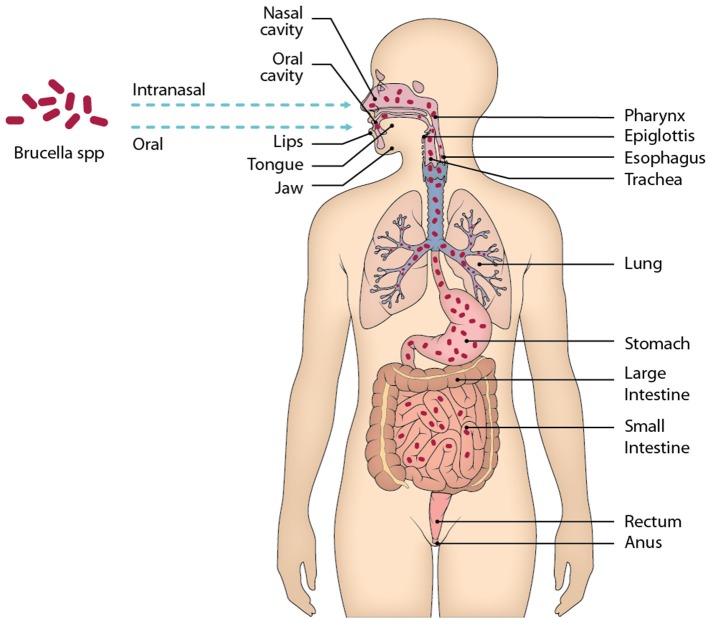
Main mucous membranes affected by the entry of *Brucella* through the oral and intranasal routes. When ingesting food contaminated with *Brucella*, the oral cavity is the first site of contact of the bacteria with the host, although it is very little time that remains there, in the oral cavity there are elements of the immune system belonging to MALT that should recognize the presence of *Brucella* and eliminate it. After the oral cavity *Brucella* could enter the gastrointestinal tract along with the alimentary bolus through the esophagus up to the stomach. In the stomach *Brucella* is apparently able to resist the pH of gastric juices, then enters the small intestine, where it will face physical and chemical barriers, as well as different cell lines and lymphoid tissue belonging to GALT. Following this route is likely to reach the large intestine and even that the bacteria was eliminated by feces, however unknown.

Although *Brucella* spp. enters the body mainly through the mucous membranes of the gastrointestinal tract and the respiratory tract, most of the anti-*Brucella* immunity studies performed so far use experimental models of parenteral infection, mainly intraperitoneal, which gives easy and rapid access to the spleen and other organs of the reticuloendothelial system (Figure 1). In this review we will discuss the concepts of innate mucosal immunity involved in mucosal protection against *Brucella* infection. The mechanisms used by *Brucella* spp. to evade the elements of oral, intestinal, and repiratory mucosal immunity will also be analyzed. In addition, the recent concept of T-cell-mediated immunity operating in the intestinal mucosa, whose characteristics differ from the systemic immunity developing in the spleen, will be discussed. The understanding of Brucella's interactions with the elements of mucosal immunity and the possibilities of experimental models should allow this information to be used to explain why this infectious disease tends to be chronic, and what will be the best vaccination strategies through these pathways.

## Oral Cavity, First Contact Site for *Brucella* spp.

The oral cavity is the first site of contact of *Brucella* spp. with the host and it is provided with an immune system mechanism belonging to mucosa-associated lymphoid tissue (MALT). Therefore, *Brucella* should initiate an immune recognition response in this tissue. The oral cavity is a highly hostile site: not only is it exposed to pathogens that enter by this route, as *Brucella, E. coli*, and *Samonella*, but it is also in constant contact with air antigens, food, or microbiota, and is subjected to mechanical damage by mastication. Consequently, the oral mucosa has defense mechanisms and tolerance similar to GALT (7, 8).

Initially, when *Brucella* enters the oral cavity, it encounters barriers such as saliva, which contains elements that eliminate or control microbial growth, such as lysozyme, lactoferrin, nystadine, peroxidases and immunoglobulins, mainly type A (IgA). Along with saliva, there is also the gingival crevicular fluid that covers the space between the teeth and the gingiva, known as the gingival sulcus. This fluid contains complement molecules, antibodies, neutrophils, and plasma cells. Therefore, the mixture of saliva and gingival crevicular fluid forms an initial strong barrier against pathogenic microorganisms (Figure 1). However, it is unknown whether *Brucella* is susceptible to this barrier or able to evade it (8).

In the mucosal tissue there are also phagocytic cells that recognize pathogens as *Brucella*. Phagocytic cells as dendritic cells and macrophages (antigen-presenting cells, APCs) able to capture antigens and migrate to the nearest regional lymph node, in this case the cervical lymph node, are distributed along the specialized tissue of the oral cavity. Once they have captured antigens from the oral mucosa, APCs migrate to the lymph node to present the antigen to the lymphocytes and send the appropriate activation signal (8, 9).

In spite of being the first contact *Brucella* has with the host, the response of the immune system against *Brucella* in this site has been poorly studied. Still, there are reports of brucellosis patients showing inflammation in the cervical lymph nodes who had apparently become infected by consuming products contaminated with *Brucella*. These findings led to the development of an animal model inducing infection with *B. melitensis* by three different routes of administration for subsequent comparison. The first one consisted of depositing the bacteria directly in the oral cavity of the mouse, the second one involved the administration of the bacteria with an oropharyngeal probe, while the third way of administration was performed intraperitoneally. It was found that, regardless of the administration route, *Brucella* infection induced inflammation of the cervical lymph node. The viable bacterium was also recovered from the first days after infection and *Brucella* was determined to persist in the GLN until 50 days after inoculation. Regardless of the administration route and because more bacteria were found in GLN than in other tissues, it is suspected that GLN may be a selective *Brucella* niche (10).

This suggests that from the first moment after ingesting food contaminated with *Brucella*, the bacterium is recognized and taken by some APCs belonging to the oral mucosa and later migrates to the cervical lymph node. Cell analysis of the inflamed cervical lymph node from the model described above indicates that there is an increase in CD68+ cells, a marker expressed by macrophages (10).

Knowledge of the passage of *Brucella* in the oral mucosa is practically unknown; for example, whether there is a participation of amygdales and/or adenoids, which are lymphoid tissue of the oral cavity. Similarly, it is unknown if the recognition of *Brucella* produces an inflammatory response in the epithelium of the oral cavity or if the response induced in the GLN extends to other sites of the host.

The oral cavity has been considered very little as the body's first point of contact with *Brucella* spp. Since the oral mucosa is the initial site of infection, more attention should be paid to the role of the lymph nodes draining to the head and neck. It is therefore possible that the oral route is a potential vaccination route.

## Interplay of *Brucella* Through the Gastrointestinal Tract

**Figure 2:**
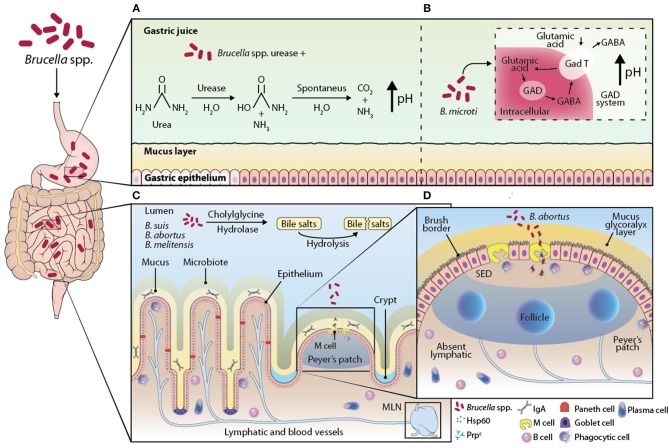
Illustration of the proposed models of the interaction of *Brucella* strains with GALT. When *Brucella* reaches the stomach, it encounters the main chemical barrier, the pH of gastric juices. Some *Brucella* spp. possess the enzyme urease, which is able to catalyze the hydrolysis of urea into carbamate and ammonia; carbamate is degraded by spontaneous hydrolysis into ammonia and carbon dioxide, creating a basic environment in the environment by increasing the pH **(A)**. It has been described in *B. microti* the GAD system that consists of transporting glutamic acid into the bacterium and with the help of the enzyme glutamate decarboxylase (GAD) convert glutamic acid into gamma amino butyric acid (GABA), this reaction consumes a proton that alkalinizes the extracellular medium of the bacterium, which would help *B. microti* survive in an extremely acidic environment such as the pH of the stomach **(B)**. The bile salts in the small intestine have an antimicrobial role, because they are capable of damaging the membrane of some bacteria, the strains *B. suis, B. abortus*, and *B. melitensis* possess the enzyme cholylglycine hydrolase, an enzyme that catalyzes the hydrolysis of bile salts, inhibiting their microbicidal activity **(C)**. In addition to the chemical barriers present in the gastrointestinal tract, there is the most important physical barrier, the monolayer of epithelial cells of the intestine. It has not been fully clarified how *Brucella* crosses this barrier, however it has been proposed that it is through M cells, specialized enterocytes that are found above the sub-epithelial dome (SED), in the epithelium region associated with the follicle. The M cells on its apical side express highly the cellular prion protein (Prpc), this protein is a receptor and one of its ligands is the Hsp60 protein, secreted by *B. abortus* through its type IV secretion system, which suggests that the interaction of the receptor and its ligand promotes the entry of the bacteria into the intestine **(D)**.

The gastrointestinal tract is daily exposed to a wide variety of innocuous antigens from foods and microbiota, and, eventually, pathogenic antigens (11–14). The integrity of the intestinal tissue depends on a local immune system consisting of physical barriers, molecules, several cell strains, and specialized lymphoid tissue associated to the intestine (Figure 2) (14, 15). The epithelium is covered with a mucus barrier that protects it against direct contact with most of the bacteria, both in the stomach and the intestine. This mucus contains a glycoprotein called mucin, which has viscous and glutinous properties to trap microorganisms that are then expelled with the aid of the peristaltic movement in the intestine (11, 15). Besides mucin, the intestinal mucosa contains proteins secreted by Paneth cells, including β-defensins, lysozyme, cathelicidins, and phospholipase A2, which act as antimicrobial agents.

On the other hand, bile salts released in the small intestine create conjugates with detergent action that damage the bacterial wall and prevent microbiota growth (11, 16). Finally, in the intestinal mucus are immunoglobulins with the capacity to neutralize antigens from intestinal lumen, mostly lgA. Cells from the intestinal epithelium are joined between them by a variety of molecules. Among these intercellular interactions are close conjunctions, whose molecules prevent antigens from entering the lamina propria via the lumen (17). The main effector site of the intestinal immune response is the lamina propria; there APCs (as CD68+ macrophages and dendritic cells, DC) are located and show an immature phenotype and high phagocytic activity. Also, polymorphonuclear cells (neutrophils, mastocytes, and eosinophils) are found in the lamina propria, especially when there is an inflammatory response. The lamina propria is also populated by T and B lymphocytes that migrate from their activation site in the lymphoid tissue to the intestine (9, 11).

The GALT is constituted by PP, isolated lymphoid follicles, and MLN. These are considered the main sites inducing the adaptive immune response since they are also the location of antigen presentation to T lymphocytes and activation of B lymphocytes, which create germinal centers (13, 17).

Given that the mouth is the natural entry for *Brucella* spp. the gastrointestinal tract is the most exposed tissue, which the bacterium uses to disseminate and start the infection, apparently, evading the local immune response. The pH of the stomach is useful to degrade proteins and is considered a chemical barrier that pathogens must confront during their transit (15). Some bacteria, as *Helicobacter pylori*, produce an enzyme with urease activity that is considered a virulence factor. This is because the enzyme hydrolyzes urea, ammonia, and carbamate, creating a basic environment in the medium. This alkaline condition promotes bacterial growth while urease byproducts provide a source of nitrogen. However, urease can compromise the integrity of the epithelial tissue (18). Urease activity could be a favorable factor to *Brucella* spp. persistence through the stomach. In the sequencing of *Brucella* spp. genome, two urease operons (*ure1* and *ure2*) were found in its chromosome 1 (19). The only *Brucella* species that does not produce urease is *B. ovis*, which is not transmitted orally but sexually in animals. This incapacity is likely to be caused by the absence of the enzyme.

The biological function of urease and its role in the pathogenesis have been evaluated in strains manipulated to eliminate operons *ure1* and *ure2*. The mutated strains were intragastrically inoculated to BALB/c mice. The researchers observed a lower colonization in the small intestine, as compared against the one observed in the wild strain *B. abortus 2308*. Still, there was no evidence that the urease-deficient strains colonize the liver or the spleen. On the other hand, in the intraperitoneal inoculation, urease-deficient strains did colonize both the spleen and the liver, which suggests that urease is a key factor, although not the only one, to secure survival through the stomach (Figure 2A) (6, 19–21).

Some bacteria such as *E. coli* and lactic acid bacteria (*Lactococcus lactis*) have a system that converts glutamic acid into gamma amino-butyric acid (GABA) inside the bacterium through the action of the enzyme glutamic acid decarboxylase (GAD) (22). This reaction consumes a proton that alkalinizes the intracellular environment of the bacterium. In addition, this system allows for a GABA molecule to be transported to the extracellular medium and a glutamic acid molecule to be internalized in the bacterial cytosol, leading to an increase in the extracellular pH. This system is primarily involved in *E. coli* resistance in extremely acidic environments, such as stomach pH. The GAD system is present in the *B. microti* strain, while the gene involved in this system is identified as *gadBC*. In the presence of a glutamic acid medium *B. microti* can grow at a pH of 2.5; however, when the *gadBC* gene is modified into *B microti* its growth in an acidic medium decrease. *In vivo*, the oral administration of these strains with the modified genes significantly decreases their CFU in spleen and liver when compared to the *B. microti* strain without the modified *gadBC* genes. Then, it is suggested that this system is a mechanism that *Brucella* can employ to resist the stomach pH (Figure 2B) (23).

The presence of *gadBC* genes has been identified with *in silico* analysis present in almost all species of *Brucella*; still, the functionality of the GAD system is not present in more pathogenic species such as *B. abortus, B. canis, B. melitensis, B. neotomae, and B. suis* (24).

Bile salts are secreted in the small intestine as conjugates with detergent properties that promote dispersion and enzymatic degradation of fats. This property also has an effect on the membrane of some bacteria; therefore, bile salts are considered to play an antimicrobial role (16). It has been described that *B. abortus, B. melitensis*, and *B. suis* synthesize an enzyme hydrolyzing bile salts, called cholyglycine hydrolase, coded by gene *chg*. This enzyme is also found in intestinal microbiota bacteria as *Lactobacillus* and *Bacteroidetes*, which suggests that microbiota bacteria require this enzyme to prevent the detergent action of bile salt conjugates (25).

The function of *Brucella* spp. cholyglycine hydrolase has been studied in strains in which gene *chg* has been previously eliminated. Studies prove the relevance of this enzyme during transit in the intestine when the knock-out strain is orally administered to mice. A decrease in the chg^−/−^ bacteria disseminated to the spleen was observed in comparison with wild bacteria that were orally administered. This study demonstrated that the viability of *Brucella* spp. transit in the gut depends on the integrity of cholyglycine hydrolase. This finding suggests cholyglycine is relevant as a virulence factor, aiding the bacteria to avoid the microbicide activity of bile salt conjugates (Figure 2C) (26).

Unlipidated 19 kDa outer membrane protein, U-Omp19, is a *Brucella* protein without lipid fraction. This modified protein is studied to be used in the development of a vaccine against brucellosis. During the studies, the sequence of this protein has been analyzed and found to be identical to the protease inhibitor proteins of other bacteria, mainly of the Inh family of *Erwinia chrysanthemi*. To demonstrate the inhibitory function of U-Omp, experiments were performed *in vitro*: U-Omp was incubated in a casein system and different proteases characteristic of the stomach (pepsin) and proteases secreted from the pancreas to the intestine (elastase, trypsin, and α quymotrypsin). The study indicates that U-Omp inhibits protease function by preventing casein degradation. The results suggest that *Brucella* can evade the proteases of the stomach and intestine of the host with this protein in order to invade the mucosal tissue and establish infection (27).

The monolayer of epithelial cells in the gut is an important physical barrier. The pathogens that reach the intestinal lumen, as *Brucella*, must cross this barrier to disseminate toward other tissues as liver and spleen. Evidence in animal models suggests that *Brucella* goes from the intestinal lumen into the small intestine and, from there, disseminates to other organs as spleen and liver (6).

The mechanism through which *Brucella* spp. is translocated from the intestinal lumen to tissue remains unclear. Still, it has been proposed that *Brucella* is introduced by enterocytes specialized in trapping lumen antigens, called M cells, which later deliver the antigen to nearby DC (6, 28). M cells are found in a region of PP, called follicle-associated epithelium. PP are distributed along the small intestine and their anatomy, cell distribution, and functions are similar to those of a node, except PP do not have capsule nor afferent lymphatic vessels (13, 29).

Studies *in vitro* demonstrated that *B. melitensis* can cross a monolayer of human epithelial cells differentiated to M cells but does not go through a monolayer of non-differentiated enterocytes, suggesting that M cells promote the transit of *Brucella* through the epithelial monolayer (6). Studies *in vivo* on the association between *Brucella* and PP were done in ligated loops, closing the ends of an intestinal section containing at least one PP where the bacterium as inoculated. These studies allowed for the observation of *B. abortus*-GFP by confocal microscopy within the follicle-associated epithelium of the Peyer's patches, a region that is rich in M cells (28). A more thorough understanding of the role M cells play was achieved studying the cellular prion protein (PrP^C^) highly expressed at the apical end of M cells. This protein is a receptor, one of its identified ligands is the heat-shock protein Hsp60, secreted by *B. abortus* through its type-IV secretion system. This suggests that the interaction between PrP^C^ and Hsp60 promotes infection by macrophages, so PrP^C^ could be a *Brucella* receptor (30). The observation that *B. abortus* inoculated in the ligated loop associated to PrP^C^ is related to the high efficiency with which the bacterium is internalized in PP. In PrP^C^-deficient mice (prnp^−/−^), a lower amount of internalized *B. abortus* internalized in the ligated PP loop was observed. However, when *Brucella* is inoculated orally in prnp^−/−^ mice, no PP were detected (31). These observations confirm the role of PrP^C^ in the capture and internalization of the bacterium by M cells, which provide *Brucella* spp. An efficient penetration into the organism (Figure 2D).

It must be noted that strains of *B. abortus, B. melitensis, B. suis, B. canis*, and *B. abortus* RB51 adhere to epithelial cells CaCo-2 and HT29 inside which they can replicate. Infection of cell lines Caco-2 and HT29 with *Brucella* strains does not induce the secretion of pro-inflammatory cytokines as TNFα, IL-1β, MCP-1, IL-10, or TGF-β; however, they produce IL-8 and chemokine CCL-20 (32). The absence of inflammatory response to the infection by *Brucella* spp. has also been observed in histological sections of the small intestine from intragastrically inoculated mice. These sections do not show neutrophil infiltration, which would be favored by IL-8 and CCL-20 secretion (6).

Despite the mechanisms mentioned on the entry of *Brucella* spp. in the organism, it is still unclear how *Brucella* avoids each barrier of the intestinal immune system. Similarly, we have yet to clarify how and why *Brucella* remains in the gut and GALT (as PP and MLN) for so long. Submandibular maxillary lymph nodes (which drain oral cavity, eyes, and nasal mucosa) can be a niche or reservoir for *Brucella* to remain silent and for periods of up to 50 days (33). This proposal could also be considered for organs as PP or MLN in which viable *Brucella* has also been found to remain for extended periods. It is also important to elucidate how *Brucella* spp. virulence factors affect innate immune response and GALT adaptive response. Once this is understood, experts will be able to develop vaccines that protect the exposed tissue of the gastrointestinal mucosa.

Although *Brucella* cannot be considered as an enteropathogen, it passes through the intestine in a natural infection. Although this has long been known to be the pathway of entry for this pathogen, most studies of pathogenesis and the immune response against *Brucella* spp. have been conducted by inoculating the bacterium intraperitoneally. This route favors systemic infection in early post-infection times, with early and easy colonization of the bacteria in the spleen. However, as it is not the natural route of infection, it does not allow knowing the immunity mechanisms that could contain the infection before its systemic dissemination. It is possible that the intestine, an effective barrier to bacterial invaders, is a possible reservoir of *Brucella* spp. For this reason, it is essential to have a detailed knowledge of the mechanisms of innate and acquired immunity that develop in the intestinal mucous membrane toward the various components of *Brucella* spp. that allow its systemic dissemination.

## Mucosal of Respiratory Tract. Other Portal of Entry of *Brucella* spp.

Oral exposure is the major route of infection and aerosol exposure is the most common source of occupationally acquired brucellosis caused by the manipulation of contaminated secretions of ill animals or by contact with laboratory cultures and tissue samples (34, 35). *Brucella* spp. can be easily aerosolized, with the additional hazard of its low infectious dose. The human infection has a variety of presentations, mainly affecting the reticuloendothelial system and complications had been reported; pulmonary involvement is sporadic (36, 37). Because of its potential spread by aerosols, *Brucella* has been classified as a biological agent of threat (38).

On the arrival of bacteria to the nasopharynx, the first step in pathogenesis is the adhesion to target molecules through different microbial ligands, most of them unknown so far. It has been reported an adhesin of *B. abortus* with an immunoglobulin-like domain. Although this protein has been involved in the adhesion to epithelial cells (39) the colonization of these bacteria in the respiratory tract has not been reported as is the case with species such as *Bordetella pertussis*, which usually enters and resides into the upper respiratory tract (40). In humans and other species, the inhalation of aerosols contaminated with *Brucella* leads to colonization of organs such as the lymph nodes, spleen, and liver (41).

The inhalation of aerosol or the intranasal instillation in mice is considered the most natural pathway of entry of *Brucella*, since the bacteria must trespass the first defense mechanisms (42). Epithelial barriers, mucus, defensins, and lysozyme avoid the colonization and entry to respiratory tract. It has been described that *Brucella abortus* PliC is a lysozyme inhibitor and it could participate in the brucellosis pathogenesis (43). Epithelial cells produce defensins, cytokines and chemokines in response to *B. abortus*. β-defensin 2 has antimicrobial activity against several pathogens and *in vitro* model β-defensin 2 does not seem to be effective against *Brucella*, but it could participate in recruiting immune cells and increase the dissemination of the bacteria (44).

In the nasal mucosa, secretory IgA and to a lesser extent IgG are other defense mechanisms. However, some evidences suggest that humoral immune response mediated by IgA may not play a central role in restraint against *Brucella* so the pathogen can even penetrate the epithelial barrier (45).

Epithelial cells and phagocytic cells are the first cells to be contacted by inhaled microorganisms. Some pathogens find temporary refuge from host immunity by sliding between epithelial cells (46). Furthermore, *Brucella* invades lung epithelial cells and alveolar macrophages where it avoids its degradation by inhibiting and modulating the endocytic traffic. In this way, these infected cells could promote the extrapulmonary dissemination of the bacteria within host tissues (47, 48). There is evidence that following intratracheal inoculation of *Brucella* in animal models, the bacteria spread to the spleen, draining cervical lymph node, tracheobronchial lymph nodes and mediastinal lymph nodes (47, 49), Therefore, immune response in mediastinal and cells homing to lungs have to be analyzed with more detail to clarify the immune responses that could mediate immune protection against mucosal infection.

Cytokines IL-1, IL-6, and TNF-α are important mediators of systemic and local inflammatory responses, but in lungs this triad do not have a key role. It has been shown that IL-1 and IL-6 deficiencies do not impact on the course of *Brucella* infection in the lungs, liver, or spleen. However, TCR-δ, TAP1, IL-17RA, and IFN-γR deficiencies affect *Brucella* control in the lungs (50). Recently, it was reported that IL-1R, probably through IL-1β action, and the NLRP3 and AIM2 inflammasomes have a protective effect on respiratory *Brucella* infection (51).

The induction of cellular-mediated responses is important in the clearance of *Brucella* from the lung, liver, and spleen, and the protection should be optimal when immunization and challenge routes are identical (45, 52). The live vaccines have shown to be effective in protection against brucellosis and these studies in animals have allowed to define the systemic immunity, but most of these studies performed parenteral vaccination and there is limited information of the mucosal response induced by the natural route of entry of *Brucella* (53).

Although no immunization route has shown total protection against intranasal challenge it has been found a significant reduction of bacterial load compared to the unvaccinated animals. Generally, it has been observed that intranasal immunization of *Brucella* induces systemic and mucosal immune responses (45, 52).

## Response of T Lymphocytes to Infection by *Brucella*

*Brucella* is one of the non-commensal pathogenic microorganisms that enter the organism through the intestinal mucosa and destabilize the commensal biota. Other pathogens such as *Escherichia coli* (ETC, EPEC, EIEC, etc.), *Shigella, Salmonella*, and *Yersinia* (54) colonize the gut and lead to the synthesis of proinflammatory molecules as TNF-α and IL-18, which are produced by macrophages in response to the infection (55).

*Brucella abortus* is captured by macrophages, where it causes the inhibition of several intracellular processes, as phagosome-lysosome fusion and respiratory burst through components as lipopolysaccharide (LPS) and those of the type-IV secretion system, which are virulence factors (6, 56–58). The most relevant virulence mechanism of *Brucella* spp. is its ability of survival and replication within these phagocytic cells as well as its mechanisms to evade intracellular death. *Brucella* spp. can infect different cell strains as macrophages, monocytes, DC, epithelial cells, and B lymphocytes (3). This characteristic is shared by other bacterial species that behave as intracellular pathogens, as *Mycobacterium tuberculosis* and *Salmonella enterica serovar Typhi* (59, 60), so the mechanisms of induced immunity are partially similar.

During the severe stage of the disease, which lasts 2 weeks approximately, the bacteria multiply rapidly. In the chronic stage, lasting 5–6 weeks, the bacterial concentration stabilizes and then decreases slowly, although there are some remaining bacteria in the liver, spleen, and lymph nodes even after 5 months (57).

The lamina propria of the intestine is inhabited by a number of cell lines, including CD4+, CD8+, γδT, and activated B lymphocytes, plasmatic cells, macrophages, eosinophils, and several populations of DC that take part in the regulation of the mucosa immune response (61). Additionally, stimulated enterocytes do have the ability to produce proinflammatory cytokines IL-1, IL-6, IL-7, and IL-8 (17).

The natural invasion of the host by *Brucella* induces a specific immune response mediated by Th1 lymphocytes that protects against the development of the disease, similar to the one observed in infections caused by other intracellular pathogens as *Salmonella* and *Mycobacterium tuberculosis*. Th1 lymphocytes are characterized by the production of proinflammatory cytokines as IL-1β, IL-6, TNF-α, and more importantly, IFN-γ (59). These proinflammatory cytokines play a protective role since they activate macrophages to increase their bactericide capacity and cytotoxic T lymphocytes (CTL) that kill infected cells (62–65). It has been observed that IFN-γ and IL-12 production by macrophages is induced by IL-17 (66). Also, the differentiation to Th17 lymphocytes is induced by a combination of IL-1, IL-6, and the transforming growth factor β (TGF-β). The *in vivo* participation of IL-23 in this differentiation process is necessary to achieve a stable differentiation and proliferation of Th17 cells when activated (67).

IL-17-producing cells, found in the lamina propria, play an essential role against microorganisms infecting the gastrointestinal tract (68). IL-17 production has also been observed in the lung and oral cavity mucosa. The cells producing the cytokine migrate toward these sites due to CCR6 expression. It has been established that IL-17 response at these mucosal sites is mainly directed against extracellular bacteria and fungi (69). Its protective role in infections caused by intracellular pathogens was initially controversial. In infections caused by *Listeria monocytogenes*, IL-17 response is produced by γδT lymphocytes in the liver, which apparently leads to the arrival of neutrophils in the organ. Nevertheless, mice lacking IL-17 receptor (IL-17RA^−/−^) survive infection by *L. monocytogenes* (70). Contrastingly, IL-17 is necessary to generate a protective immune response against *M. tuberculosis* induced by vaccination.

The role of IL-17 seems to be differentially relevant with respect to *Brucella* species, against which protection is created. In oral vaccination of BALB/c mice with *B. melitensis* strain from which gene *znuA* (involved in zinc transportation) or *B. abortus* RB51 strain induced a similar protection against the intranasal challenge with *B. melitensis* 16M. The mice that showed IFN-γ deficiency (IFN-γ^−/−^) also exhibited a state of protection, albeit less significant than the one observed in BALB/c wild-type mice. The protection in both mice strains was parallel to IL-17 and IL-22 response. However, IL-17 and IL-22 production was higher in IFN-γ^−/−^mice than in wild type BALB/c mice, which would suggest that IL-17 and IL-22 compensate for the absence of IFN-γ. The *in vivo* neutralization of IL-17 with specific antibodies only affected the protection conferred by strain RB51 but did not affect the one provided by mutant Δ*znuA* (71). This proved that IL-17 is relevant to induce the protection provided by strain RB51 when administered orally.

Other studies have demonstrated the role that IL-17 plays in the protection against brucellosis induced by vaccination in the intestinal mucosa (72). *B. abortus* inhibits the host's proteases through Omp19 and limits the antigenic proteolysis of cells from PP and MLN, increasing the bacterial concentration in the gut mucosa inside monocytes and DC. However, in mice immunized with ovalbumin together with *Brucella* U-Omp19 as adjuvant, the anti-ovalbumin response of Th1, Th17, and CD8+ T lymphocytes was increased in the gut mucosa and system, leading to a high production of IFN-γ and IL-17 (27).

Abkar et al. (73) orally immunized BALB/c mice with protein U-Omp19 incorporated into N-trimethyl chitosan (TMC/Omp19) nanoparticles. They compared the response with that observed in mice immunized with intraperitoneal TMC/Opm19 after being challenged with virulent *B. abortus* strains 544 and *B. melitensis* 16M. The results showed that vaccination with oral TMC/Opm19 generated a protection considerably higher than the one induced by intraperitoneal vaccination. In addition, the intraperitoneal vaccination induced Th1- and Th2-type responses while the oral vaccination induced Th1 and Th17 cell response. These results demonstrate that the oral presentation of *Brucella* spp. antigens induce a more efficient response in which IL-17 production is deeply involved.

Lymphoid aggregates (particularly γδT lymphocytes, cryptopatches) have been identified in the lamina propria of mice. In these aggregates, Tγδ and CD8+αα lymphocytes suffer extrathymic maturation (74). Only a few studies on the role of γδT cells have been reported; however, it is well-known that they are relevant in the early stages of infection since TCRδ^−/−^ mice are more susceptible to infection by *B. abortus* than wild-type C57BL/6. During the first week post infection, there was an increase in γδT cells, which abated after 2 weeks. On the other hand, IFNγ^−/−^ mice, from which γδT cells were eliminated, showed a considerable increase in susceptibility to the infection by *B. abortus* (75).

In contrast, CD4+CD25+ Tregs cells are involved in the early stages of infection by *Brucella*, reducing the capacity of response of CD4+ effector T cells. The elimination of CD4+CD25+ Tregs cells with anti CD25 antibodies triggered an enhanced protective response against *B. abortus* in BALB/c mice, along with an increase in IFN-γ production compared with the response of mice without anti CDC25 treatment. In persistent infection, CD4+CD25+ Tregs cells maintain their suppressor function (76).

At the beginning of the infection by *B. abortus*, the number of Tregs and effector T cells was increased (76, 77). In this early stage of the infection, Tregs can negatively regulate the function of effector T cells through the production of IL-10 (78). Splenocytes can also produce IL-10 after the stimulus by PrpA protein of *B. abortus* and thus promoting the persistence of the infection (79). This suggests a deleterious role of the anti-*Brucella* immune response mediated by Treg and IL-10 production.

It is remarkable that in other infectious processes, CD4+CD25+ Treg and IL-10 protect against the infection or the disease. This has been observed in the tuberculosis murine model (80), and in infections caused by *Helicobacter pylori* (81), *Pneumocystis carinnii* (82), or trypanosomiasis (83).

It is well-established that the development of Th1 cell-mediated immune response is important in protection against *Brucella* spp. Particular emphasis has been placed on the role of IFN-γ in protecting against infection caused by *Brucella* spp. This conclusion has been drawn from studies done on animals infected by parenteral pathways, in which a systemic infection develops directly. Protective immunity in the intestine continues to be dependent on T cells, but it has become clear that other subpopulations are involved. In addition, a delicate balance must be maintained in the gut between pro-inflammatory, classically anti-*Brucella*, and anti-inflammatory responses. This knowledge is required for the design of vaccines that must be applied through the digestive tract to generate local immunity in the intestinal mucosa.

## Antigenic Components of *Brucella* as Virulence Factors

*Brucella* spp. is an intracellular pathogen that invades and proliferates within the host's cells. The virulence of this strain is associated to the intracellular replication capacity in phagocytic and non-phagocytic cells (84). *Brucella* spp. lacks the typical virulence factors as toxins, fimbria, pili, and plasmids (85). Contrastingly, this intracellular pathogen has different antigenic elements that provide it with virulence and allow it to establish in the host's cells.

### Lipopolysaccharide

*Brucella*, as genus, has two colonial morphologies: smooth and rough. In general, *Brucella* strains of rough morphology are attenuated or show reduced virulence, with the exception of species *B. ovis* and *B. canis*, that are virulent and naturally rough. The LPS of *Brucella* is one of its main virulence factors and is considered non-conventional when compared against other Gram-negative LPS, as those from the *Enterobacteriaceae* family. Neither *B. abortus* LPS nor that of *B. melitensis* activate complement or macrophages while their endotoxic activity is extremely reduced (86, 87).

The LPS of *Brucella* spp. is constituted by three main components: lipid A, core, and O side chain, which is the immunodominant component. Lipid A is inserted in the external membrane and has a backbone of diaminoglucose; acyl groups are the longest chain, C18–C19 or C29 instead of C12 to C14 of conventional LPS. The core, to which acylated chains are bound, has a polysaccharide sequence conserved among *Brucella* species. The bond between the acylated chain and core is through an amide linkage instead of ester and amide linkages. The O side chain of smooth LPS from *Brucella* is a chain of repeating units of sugars with a variability that allows for the differentiation of the species (88).

LPS from *Brucella* exhibits low endotoxicity due to poor macrophage recognition by heterodimer TLR-2/MD2, considered the main LPS receptor in Gram-negative bacteria that also transduces activation signals within macrophages. In consequence *Brucella* LPS does not induce a response in macrophages or DC *in vivo* or *in vitro*. The stimulation of macrophages or DC with *Brucella* spp. LPS does not induce the expression of activation markers on the cell surface nor does it induce the production of proinflammatory cytokines. In contrast, *Brucella* spp. LPS seems to deregulate macrophages and DC according to their function of antigen presenting cells, so it does not allow for the activation or proliferation of T lymphocytes. This low induction in the immune response mediated by cells could be the way in which *Brucella* evades from the beginning of the infection. It allows *Brucella* to establish, triggering the chronicity of the infection (89).

It has been demonstrated that the core is a virulence factor, since wadC mannosyl transferase mutants increase interaction with MD2, leading to a higher production of cytokines. On the other hand, the loss of the O-chain creates attenuated strains that allow the infected cell to carry the vesicle containing *Brucella* to the lysosomal compartment, where the bacterium is eliminated. Smooth strains evade from this intracellular death pathway, suggesting that the O side chain of LPS is involved in the transportation of the vesicle containing *Brucella* to other non-lysosomal compartments, or avoidance of transport to such lysosomal compartment (90). *Brucella* spp. LPS also prevents the activation of the complement, with which it also avoids opsonization by subcomponent C3b, decreasing recognition by phagocytic cells through receptors for the complement. Additionally, it prevents the generation of anaphylatoxins C3a and C5a, hampering the consequent proinflammatory response (86, 87).

### Cyclic β-1,2-Glucan

Cyclic glucans are intrinsic components of Gram-negative bacteria that have a high structural variability and are responsible for maintaining homeostasis against osmolarity changes (91). *Brucella* spp. cyclic glucans are accumulated in the periplasm and are constituted by a backbone of glucose units bound by b-1,2 links with a polymerization degree of 17–25 monomers (92). Mutants of the genes responsible for the synthesis and transport of glucan to the periplasm show decreased survival and multiplication of the bacterium in BALB/c mice spleen. The mutant strain of the gene *cgs* (cyclic glucan synthase), responsible for glucan synthesis induces a response that is almost exclusive of Th1 lymphocytes (93). It has been demonstrated that the cyclic glucan of *Brucella* spp. acts upon the host cell membrane at the lipid rafts. Then, the bacterium controls the maturation of the vacuoles in which it is internalized, preventing lysosome fusion, and allowing to reach the replication niche (94). The cyclic β- 1,2-glucan of *Brucella* spp. promotes splenomegaly in mice as a consequence of monocyte, DC, and neutrophil recruitment by IL-12 and TNF-α induction (95). Studies *in vitro* have demonstrated that *Brucella* spp. induces DC activation via TLR4, MyD88 and TRIF, but not through CD14, increasing the antigen-specific response of CD8+ T lymphocytes (96).

### Type IV Secretion System and BvrR/BvrS

*Brucella* spp. has a two-component system BvrR/BvrS. This system directly and indirectly controls the expression of operon virB, coding the type IV secretion system (T4SS) (97). The two-component BvrR/BvrS system of *Brucella* spp. is constituted by a histidine-kinase sensor located in the cytoplasmic membrane (BvrS) and a cytoplasmic regulator (BvrR). The mutant strains in the BvrR/BvrS system are avirulent in mice, show a lower capacity to invade macrophages and HeLa cells and are unable to replicate intracellularly (98).

The *Brucella* BvrR/BvrS two-component regulatory system is essential to detect changes in the phagosomal environment. In addition, it allows the bacterium to modify the lifestyle from extracellular to intracellular (99). The BvrR/BvrS system controls the conformation of the LPS structure and the expression of Omp (99–101). This system is also necessary to switch off extracellular survival genes and express the genes needed for invasion and intracellular survival (97).

The *Brucella* T4SS, constituted by 12 components, has been evaluated using *in vitro* and *in vivo* models. The *in vitro* infection of different cell lines demonstrated that the T4SS is essential to allow for the intracellular replication of the bacterium. In animal models it has been observed that T4SS is necessary for the onset of the infection (102). Recently it has been published that BvrR is a good candidate for a DNA vaccine in the murine brucellosis model, but many studies are missing (103).

### Enzyme Superoxide Dismutase

The survival of *Brucella* within the macrophage has been associated to the synthesis of several proteins: antioxidant enzymes as KatE and Cu,Zn superoxide dismutase (Cu/Zn SOD). Superoxide dismutases (SODs) are metalloenzymes that catalyze the dismutation of superoxide ions, being an important mechanism of antioxidant defense (104). Periplasmic SODs are a key element of the defense mechanisms several pathogens must protect against the respiratory burst of the host's phagocytic cells. This is a previous survival step that allows for the later replication of the bacteria within phagocytes (105).

*Brucella* spp. Cu/Zn SOD is located in the periplasmic space of the cell wall. The bacterial count of *B. abortus* Cu/Zn SOD mutants recovered from BALB/c mice is much lower when compared to the amount recovered from wild *B. abortus* (106). This result demonstrated the key role that Cu/Zn SOD plays in the virulence of the bacterium.

### Urease

This enzyme catabolizes the hydrolysis of urea in carbon and ammonic dioxide. Urease has been reported as a virulence factor for many pathogens, which induce direct toxicity in renal tissue and the formation of kidney stones. It allows for the transit of microorganisms as *Yersinia, Klebsiella*, and *Helicobacter pylori* to colonize the stomach and promotes the bacterium in an acid environment. Sangari et al. (21) evaluated urease operon mutants that were efficiently eliminated, evidencing that the presence of the enzyme promotes its survival and thus promotes infection.

## Vaccines

The most effective strategy to prevent the spread of brucellosis in humans, besides the pasteurization of dairy products, has been the control of cattle through vaccination. Although there are effective vaccines to control brucellosis, the disease has not been eradicated from most of the countries around the world (107). The available vaccines present several disadvantages as the interference with the immune response induced by diagnosis methods; some cause abortions in pregnant animals while the immunity they generate does not protect the cattle vaccinated throughout their reproductive life (107, 108). To date, there is no vaccine for safe application in humans since it is considered that even those strains known for their stability can revert their attenuated state (they must be applied as live vaccine) and trigger the disease in the vaccinated population. Vaccination programs mostly focus on females because it has been suggested that some vaccines may damage the male reproductive system (109).

According to Ko and Splitter (84), Dorneles et al. (107), Nicoletti (110), and Schurig et al. (111) the ideal vaccine against brucellosis must have the following characteristics: (1) it must be constituted by living bacteria able to generate a potent Th1-type response; (2) it must not induce the production of antibodies that may interfere with diagnosis tests to detect infected animals, regardless of the route or dosage and age of animals; (3) it must be an attenuated strain that does not cause disease nor persistent infection in animals; (4) it must not be pathogenic for humans, preventing accidental contamination during the administration of the vaccine; (5) it must induce long-term protection with only one dose, without causing abortions even when administered to pregnant females; (6) it must not induce antibody response when applying boosters; (7) it must be stable and not revert to its virulent state *in vivo* or *in vitro*; and (8) it must be affordable for its massive application and easy to produce and administer (Table 1).

**Table 1:** Ideal *Brucella* vaccine (107).

1. Constituted by living bacteria able to generate a potent Th1-type response.
2. Not induce the production of antibodies that may interfere with diagnosis tests to detect infected animals.
3. An attenuated strain that does not cause disease nor persistent infection in animal.
4. Not be pathogenic for humans, preventing accidental contamination during the administration of the vaccine.
5. Induce long-term protection with only one dose, without causing abortions even when administered to pregnant females.
6. Not induce antibody response when applying boosters.
7. Stable and not revert to its virulent state *in vivo* or *in vitro*.
8. Affordable for its massive application and easy to produce and administer.

Most of the vaccines currently used aim to prevent the disease caused by *B. abortus* (strains 19, RB51, 45/20, and SR82) and *B. melitensis* (strain Rev. 1) (108, 112). Vaccine strains for *B. suis, B. ovis*, and *B. canis* have been developed experimentally without reaching massive application. Table 2 summarize the classification of Brucella vaccines.

**Table 2:** Classification of *Brucella* vaccines.

**Classification**	**Vaccine**	**Route of administration**	**Immunological parameter evaluated**	**References**
Vaccines from recombinant strains	*RB51WboA* *RB51SOD* RB51SOD/WboA	Intraperitoneal in BALB/c mice	Concentration of IFN-γ in culture supernatants of splenocytes upon *in vitro* stimulation. Clearance of challenge infection with *B. abortus* 2308 and *B. melitensis* 16 M measure as CFU in spleen in mice previously vaccinated with mutant strains. Development of IgG2a: *RB51SOD*, developed antibodies to SOD *RB51WboA*, develop to the O-side chain RB51SOD/WboA, develop to SOD and the O-side chain.	(113–117)
	Mutants in genes *wbkA* and *per*	Intraperitoneal in BALB/c mouse	Clearance of challenge infection with *B. abortus* 2308 measure as CFU in spleen in mice previously vaccinated with mutant strains.	(118–120)
	*S2308DATP*	Intraperitoneal in BALB/c mouse	Clearance of challenge infection with *B. abortus* 2308 measure as CFU in spleen and blood in mice previously vaccinated with mutant strains. Evaluation in the expression of MCH I, MHC II and costimulatory molecules CD40, CD80 and CD86. IgG evaluation.	(121)
	*B. abortus Δpgk*	Intraperitoneal BALB/c, 129/Sv, C57BL/6, and IRF-1 KO mice	Clearance of challenge infection with *B. abortus* 2308 measure as CFU in spleen in BALB/c, 129/Sv, C57BL/6, and 129/Sv mice previously vaccinated with mutant strains, *B. abortus* S19, RB51. IFN-γ production by spleen cells of IRF-1 KO mice vaccinated with S19, RB51, or the Δpgk mutant strain.	(122)
	*S19ΔvjbR*	Vaccination intraperitoneally BALB/c mouse with a sustained-release vehicle to enhance vaccination efficacy was evaluated utilizing the live S19ΔvjbR::Kan in encapsulated alginate microspheres containing a non-immunogenic eggshell precursor protein of the parasite *Fasciola hepatica*	Clearance of challenge infection with *B. abortus* 2308 measure as CFU in spleen in mice previously vaccinated with S19 and mutant strains encapsulated and non-encapsulated.	(123, 124)
		IRF-1^−/−^ mice were vaccinated intraperitoneally with *B. abortus* S19ΔvjbR	Clearance of challenge infection with *B. melitensis* 16M measure as CFU in spleen in mice previously vaccinated with S19ΔvjbR	(125)
	*ΔznuA*	Intraperitoneal in BALB/c mouse	Clearance of challenge infection with *B. abortus* 2308 measure as CFU in spleen in mice previously vaccinated with S19, RB51 and mutant strains.	(126, 127)
Probiotic vector	*Lactococcus lactis* expressing antigen L7/L12 of *B. abortus*	Intragastric gavage in BALB/c mouse	Evaluation of fecal anti-L7/L12 IgA and systemic IgG anti-L7/L12. The mutant strain was challenged by intraperitoneal injection with *B. abortus* 2308, and the clearance in the spleen was measure.	(128)
	*Lactococcus lactis* expressing Cu, Zn superoxide dismutase (SOD) of *B. abortus*	Oral in BALB/c mouse with *L. lactis* expressing Cu/Zn alone or in combination with *L. lactis* expressing IL-12	Evaluation of the presence IgG1, IgG2a, IgM, and sIgA from nasal and bronchoalveolar lavages. Lymphocyte proliferation assay after oral immunization. The mutant strain was challenged by intraperitoneal injection with *B. abortus* 2308, and the clearance in the spleen was measure.	(129)
Bacterial vector:	*Salmonella typhimurium* expressing BCSP31, Omp3b, and SOD proteins of *Brucella abortus*	Intraperitoneal and oral in BALB/c mouse	Evaluation of IgG, TNF-α, and IFN-γ. The mixture of mutant strains was challenged by intraperitoneal injection with *B. abortus* 544, and the clearance in the spleen was measure.	(130)
	*Salmonella typhimurium* expressing BLS, Omp19, prpA, and SOD proteins of *Brucella abortus*	Intraperitoneal in guinea pigs	Histopathological assessment in lungs, liver, spleen, and uterus. The mixture of mutant strains was challenged by intraperitoneal injection with *B. abortus* 544, and the clearance in the spleen and liver was measure.	(131)
		Intraperitoneal in pregnant guinea pigs	Histopathological assessment in lungs, liver, spleen, and uterus. The mixture of mutant strains was challenged by intraperitoneal injection with *B. abortus* 544, and the clearance in the spleen and liver was measure.	(132)
Attenuated strains	*B. neotomae*	Intraperitoneal in BALB/c mouse with *B. neotomae* and *B. neotomae* mutant strains	Levels in serum of total IgG, as well as IgG1, IgG2a, IgG2b, IgG3, and IgM. IL-2, GM-CSF, IFN-γ, TNF-α, IL-4, IL-5, IL-10, IL-12p70 cytokines were tested in the collected supernatants of splenocytes from vaccinated mice. Cells from spleens of vaccinated mice were stained, to determine the proportion of CD4+ and CD8+ T cells that secreted IFN-γ. *B. neotomae* and *B. neotomae* mutant strains were challenged by intraperitoneal injection with *B. melitensis* 16M*, B. abortus* 2308, or *B. suis* 1330, and the clearance in the spleen was measure.	(133)
		Oral in BALB/c mouse	Levels in serum of total IgG, as well as IgG1, IgG2a, IgG2b, IgG3, and IgM. Levels in intestinal secretions of total IgG, IgM, and IgA. Cells from spleens of vaccinated mice were stained, to determine the proportion of CD4+ and CD8+ T cells that secreted IFN-γ and TNF-α. *B. neotomae* was challenged by intraperitoneal injection with *B. abortus* 2308 and the clearance in the spleen, liver and lungs was measure.	(134)
	*znuA B. melitensis*	Oral in BALB/c mouse and IFN-γ^−/−^ BALB/c mouse	Evaluation for colonization in spleens, Peyer's patches, and mesenteric lymph nodes (MLNs). Splenocyte production of IFN-γ, IL-17 and IL-22, was evaluated, pre and post challenge. Vaccination with *znuA B. melitensis* and *B. abortus* RB51, was nasally challenge with *B. melitensis* 16M. Clearance in the spleen and lungs was measure. Spleen grown was measure.	(71)
			Evaluation of Lc T CD4+ and CD8+.	(135)
	*B. melitensis WR201*	Oral in BALB/c mice	Vaccination with the mutant strain was nasally challenge with *B. melitensis* 16M, clearance in the spleen, liver and lungs was measure. Determination of IgG and IgM in serum and IgA in saliva. Splenocyte cytokine production of IL-2 and IFN-γ.	(136)
Viral vectors	Influenza virus vectors expressing proteins Omp16, Omp19, SOD, or L7/L12	Pregnant sheeps and goatsSubcutaneous and conjunctival routes	Serum samples for determine antigen-specific humoral IgG, IgG2a, IgG1 antibodies, and whole blood for T cell stimulation index and IFN-γ production.	(137)
			Challenged with a virulent strain of *B. melitensis*16M, concentration of the bacteria in lymph nodes (submandibular, retropharyngeal, right subscapular, left subscapular, mediastinal, bronchial, portal, para-aortic, pelvic, mesenterican dudder), parenchymal organs (liver, kidney, spleen, and bone marrow) and placenta.	
Cell subunit vaccines	BLSOMP31	Conjuntival in lambs	Samples of serum, saliva, nasal, preputial and lacrimal secretions for detection of IgG. Samples of saliva, nasal, preputial and lacrimal secretions for detection of IgG and IgA anti-BLSOmp31 levels. IFN-γ in blood samples. Intradermal reaction to BLSOmp3.	(138)
Non-pathogenic alphaproteobacteria (NPAP) antigens	*Ochrobactrum anthropi, Sinorhizobium meliloti, Mesorhizobium loti, Agrobacterium tumefaciens*	Subcutaneous, intraperitoneal and Intragastric in BALB/c mouse	The subcutaneous and intraperitoneal vaccine administration was challenge intravenously with *B. melitensis* H38. Clearance in the spleen was measure. The intragastric vaccine administration was challenge with *B. abortus* 2308. Clearance in the spleen was measure. Serum IgG against *B. abortus* cytosolic antigens in mice immunized intraperitoneally with cytosolic fractions of NPAP. Serum IgG and IgA and fecal IgA against *B. abortus* cytosolic antigens in mice orally immunized.	(139)
Nanoparticles	Omp31-loaded N-trimethyl chitosan nanoparticles	Intraperitoneal and oral in BALB/c mouse	Determination of IgG1 and IgG2a and IgM in serum. Anti-Omp31 IgA was determined in fecal samples. Cytokine (IFN-γ, IL-12, IL-4, and IL-17) response in splenocytes. The vaccine was challenge with *B. melitensis* 16M, clearance in the spleen was measure.	(140)
Outer membrane vesicles (OMVS)	(OMVs) of *B. melitensis* 16M	Intramuscular in BALB/c mouse	Serum immunoglobulin IgG1 and IgG2a isotypes with specificity to OMVs. Cytokine response of Bone Marrow-Derived Dendritic Cells (BMDC) from Balb/c mouse, Th1 (IFN-γ, IL-2, IL-6, IL-12, and TNF-α), DC2-mediated Th2 (IL-4 and IL-10), and DC17-mediated Th17 (IL-17, IL-23, and TGF-β). Mice were challenge intraperitoneal with *B. melitensis* 16M, clearance in the spleen was measure.	(141)

## Live, Attenuated Vaccines

These vaccines are given parenterally and generate immunity against systemic infection. However, as previously discussed, local mucosal immunity can precisely prevent systemic spread. It should be studied whether the use of live vaccines could maintain long-lasting immunity in the gastrointestinal and/or respiratory mucosa. Developing and maintaining local immunity will be important, as some evidence suggests that mucous membranes are reservoirs of *Brucella* spp.

### *B. abortus* S19

This strain was originally isolated from milk in 1923. It was accidentally attenuated after keeping it at room temperature for a year. Its use as vaccine in cattle started in 1941 (108, 111, 142). Strain S19 is indicated for its application in calves since it causes epididymitis in male adults and is associated to abortion in pregnant females (143). Animals vaccinated with *B. abortus* S19 develop antibodies against LPS because the strain is smooth. Therefore, it is not possible to differentiate between animals vaccinated with this strain and naturally infected animals (108, 144). Recently, it was noticed that immune response is different among breeds of cattle, as the Shawial breed responded less to the 19S vaccine than the crossbread animals (145).

### *B. abortus* 45/20

This strain was derived from an isolated smooth strain obtained from an infected cow (called 45) in 1922. After passages through guinea pig, strain 45 was obtained and, after 20 passages, the rough phase was isolated; therefore, the strain was named 45/20. This strain had to be administered inactivated by heating along with an adjuvant. It can revert to its virulent smooth form, so it has to be inactivated before applying it as vaccine (108, 111). Unfortunately, the protection and antibody response assays showed high variability, which questioned the efficacy of the vaccine. Additionally, some animals exhibited hypersensitivity reactions on the injection site of the vaccine. These inconveniences led to the interruption in the use of this strain as vaccine (107).

### *B. abortus* RB51

Smooth strains commonly show phase variation into rough strains. This change in phenotype is usually accompanied by a decrease in the strain's virulence. However, some species are naturally rough, as *B. ovis* and *B. canis*. These last are pathogenic for a natural host, but the number of animal species they infect is reduced when compared against the diversity of hosts where smooth strains can be isolated. Based on these observations and the experience creating strain 45/20, experts began looking for a notably attenuated rough mutant that could colonize the host for the time needed to induce a protective immune response (111).

The strain *B. abortus* RB51 was obtained after successive passages of the virulent smooth strain *B. abortus* 2308 in a culture medium with rifampicin or penicillin. Strain RB51 shows a rough phenotype and lacks the LPS polysaccharide O-chain. It is highly stable and does not revert to the virulent smooth phenotype (142). The absence of O-chain does not induce the formation of antibodies, which allows for the differentiation between vaccinated and naturally infected animals (107). In contrast, the strain induces a strong Th1-type cell response, which confers a high protective efficiency. This effectiveness is due to the induction of IFN-γ response, which leads to the activation of cytotoxic T lymphocytes, which eliminate the infected cells, and increase the bactericide activity of macrophages (143). In 1990, the use of strain RB51 as vaccine of choice in the USA was implemented to control bovine brucellosis and is currently applied in several Latin American countries (108). The fact that it was selected as rifampicin-resistant is a considerable disadvantage since it is the first-choice antibiotic against brucellosis when streptomycin cannot be administered (146).

### *B. abortus* SR82

Strain SR82 was developed in the former Soviet Union and was first applied as vaccine for cattle in 1988. It is recognized by the R and S phase antisera. It is as efficient as vaccine S19 but does not induce false positives in serological diagnosis tests because the presence of antibodies against R phase can be evaluated (107, 147).

### *B. melitensis* Rev1

Strain Rev1 is smooth, behaves as attenuated strain, and is applied to goats and sheep. It was derived from *B. melitensis* 16M and requires the addition of streptomycin to the medium for its growth. This requirement is a disadvantage since streptomycin is the first-option antibiotic for brucellosis treatment, usually administered together with tetracycline (109, 111, 146); its administration is preferably conjunctival. This strain also induces the production of antibodies that interfere with the diagnosis: vaccinated animals cannot be differentiated from those that are naturally infected (109).

Vaccination with live strains has proven to have a highly protective efficiency in cattle and wildlife animals. This is because the bacterium remains viable in the host for a certain time and replicates. This promotes an immune response mediated by T lymphocytes against components of the pathogen, guaranteeing its effectiveness as protection-inducing agent against brucellosis (84, 148).

## Mucosal Vaccines

The mucus membranes, covering the intestinal and urogenital tracts, conjunctiva, auditory canal, and exocrine gland ducts, have a highly specialized mucosal immune system that protects these surfaces against external attacks (149, 150). The design of vaccines that activate immunity in the mucus membranes seem to be promising. However, for a vaccine to be successful, it must be absorbed by the intestinal epithelium and captured by antigen-presenting cells in the intestine. It must also induce local response of B and T lymphocytes but must not create tolerance (151). Vaccines that are applied in the mucosa, particularly those that are orally administered, face dilution by mucous secretion; they may be trapped in the mucus and excluded with it or degraded by mucosal enzymes (152).

## Probiotic Vectors

Several authors have explored the use of lactobacilli as vectors to generate oral vaccines. This possibility has been explored since probiotics easily survive in the gastrointestinal tract without hurting it, maintaining a close association with the epithelium. In addition, some authors have studied their immunomodulatory properties (153, 154). Because of this last property they have been applied together with vaccines for intracellular organisms. For instance, *L. rhamnosus* is adjuvant for vaccination with polymorphic membrane protein C (N-Pmpc) administered in the conjunctiva and increases the humoral and specific cell immune responses (155).

The following experiences have been documented when probiotics are applied together with vaccines to prevent brucellosis.

### Lactococcus Lactis Expressing Antigen L7/L12 of *B. abortus*

L7/L12 is a ribosomal protein inducing cell-type response. Ribeiro et al. (156) transformed *L. lactis* using gene L7/L12. The vaccine administered orally in BALB/c mice induced significant lgA levels in feces against L7/L12, but no specific antibodies were found in serum; therefore, a systemic response was not triggered. The intraperitoneal application of the probiotic created a better protection (128).

### Lactococcus Lactis Expressing Cu, Zn Superoxide Dismutase (SOD) of *B. abortus*

Bermúdez-Humarán et al. (157) showed that when administered in C57BL/6 mice by nasal route, *L. lactis* transformed to produce IL-12 in conjunction with *L. lactis*, and E7 antigen of the human papillomavirus type 16 (human papillomavirus type 16 E7 antigen) presented a Th1-type response induced with significant secretion of IFN-γ in splenocytes. Based on this study two recombinant strains of *L. lactis*, one expressing Cu/Zn SOD and another expressing IL-12, were administered to BALB/c mice orally. The animals produced significant SOD-specific slgA in nasal and bronchoalveolar lavage and T cell proliferation as a response to re-stimulation with SOD or crude *Brucella* protein extract. Protection against challenge with *B. abortus* 2308 was similar to the one observed in mice vaccinated with RB51 and it was better when administered together with the two recombinant *L. lactis* strains (129).

## Bacterial Vectors

### *Salmonella typhimurium* Expressing BCSP31, Omp3b, and SOD Proteins of *Brucella abortus*

BALB/c mice were intraperitoneally and orally immunized with the mix of *S. typhimurium* recombinant strains. The concentration of lgG, TNF-α, and IFN-γ in serum of mice immunized in both routes (except the recombinant one expressing Omp3b, orally administered) was significantly higher when compared against the control inoculated with wild-type *S. typhimurium*. Protection against challenge with intraperitoneal *B. abortus* 544 was higher in mice vaccinated with the mix of recombinant bacteria administered orally (130).

### *Salmonella typhimurium* Expressing BLS, Omp19, prpA, and SOD Proteins of *Brucella abortus*

A vaccine using an attenuated *S. typhimurium* strain was designed and proved to be well-tolerated when administered orally or subcutaneously; it induces low production of TNF-α and lower cytotoxicity (158). *S. typhimurium* transformed to express Cu/Zn SOD, lumazine synthase (BLS), Omp-19, and proline racemase protein A (prpA) (131, 158) induces a good level of protection against challenge with *B. abortus* 544 when orally administered. This protection correlated with Th1 and Th17 response (72).

In addition, researchers have conducted experiments in which the vaccine additioned with *B. abortus LPS* is intraperitoneally administered to guinea pigs. Protection against challenge with *B. abortus* 544 was acceptable and did not generate histopathological damage in lungs, liver, spleen, and uterus (132, 159).

## Attenuated Strains

### B. neotomae

One of the options evaluated in the creation of new vaccines against brucellosis is the use of *Brucella* strains that are non-pathogenic for humans or cattle. One of these naturally attenuated strains is *B. neotomae*, a smooth strain isolated from desert mice.

Moustafa et al. (133) designed four vaccine strains from *B. neotomae: B. neotomae* irradiated with gamma rays, which maintains the bacterium metabolically active but prevents its replication, *B. neotomae* SOD, and *B. neotomae* Bp26 (26 kDa). The strains were intraperitoneally administered to BALB/c mice that were challenged with *B. abortus* 2308, *B. melitensis* 16M, and *B. suis* 1330. The highest protection was provided by the irradiated strain, which also induced the production of IFN-γ, IL-12p70, IL-5, and IL-10. Based on this result, Dabral et al. (134) orally vaccinated BALB/c mice with the irradiated *B. neotomae* strain and *B. abortus* RB51 as reference. In both groups of mice, antigen-specific CD4+ and CD8+ T cells secreting IFN-γ and TNF-α were induced. The challenge with intraperitoneal and intranasal *B. abortus* demonstrated that protection induced by *B. neotomae* was better than the one by RB51.

### znuA B. melitensis

Yang et al. (126) demonstrated that *B. abortus* strain znuA showed a deficiency while transporting zinc but generated a good protective response. They generated the same gene deletion in *B. melitensis* and orally vaccinated BALB/c mice that were later challenged nasally with *B. melitensis* 16M. The vaccine induced Th1 response in the spleen and respiratory lymph nodes, inducing 10-fold more CD8+ T lymphocytes producing IFN-γ than CD4+ T lymphocytes. Additionally, a systemic and mucosal Th-17 response was generated (71, 135). Two weeks after the administration, no colonies of the mutant strain were detected in spleen, PP or MLN. In contrast, mice immunized with RB51 and S19 still showed the bacterium in the organs after 2 weeks. This result indicates that *B. melitensis znuA* is less virulent (160, 161).

### *B. melitensis* WR201

This is a purine-auxotrophic strain harboring operon *purEK* deletion. The orally administered bacteria are no longer isolated from spleen, lungs, and liver 8 weeks after immunization. It induces a good protection against nasal challenge with *B. melitensis* 16M (136).

## Viral Vectors

Influenza virus vectors expressing proteins Omp16, Omp19, SOD, or L7/L12 were administered subcutaneously and through the conjunctiva along with the adjuvant Montanide™ Gel 01 in goats and sheep simultaneously. The vaccination induced 70% protection in pregnant animals against challenge with *B. melitensis* 16M (137).

## Cell Subunit Vaccines

### BLSOMP31

Decameric *Brucella* lumazine synthase (BLS) has been proven to be a protein able to carry antigens without altering their conformation due to its physicochemical and immunogenic properties. In addition, it induces activation of CD8+ T lymphocyte response and IFN-γ production (162). Estein et al. (163) used BLS and added an exposed loop of Omp31 and proved that this combination induced protection against challenge with *B. ovis* administered parenterally to goats. The same antigen was administered at the conjunctiva to 5-month-old muttons, using a thermoresponsive and mucoadhesive gel and chitosan as carriers (BLSOmp31-P407-Ch). The antigen generated specific lgG- and lgA-specific response, increased IFN-γ in serum, and development of an intradermal reaction at 90 days post infection (138).

## Alphaproteobacteria

Since it has been proven that there is a cross-reactivity between *Brucella* and non-pathogenic alphaproteobacteria (NPAP) antigens, Delpino et al. (26) vaccinated BALB/c mice with *Ochrobactrum anthropi, Sinorhizobium meliloti, Mesorhizobium loti, Agrobacterium tumefaciens*, or *Brucella melitensis* H38 as positive control. The best response to the intravenous challenge with *B. abortus* 2308 was found in mice vaccinated with *O. anthropi, M. loti*, and *B. melitensis* H38, compared against the response in mice administered PBS and that in mice vaccinated with cytosolic extracts.

Delpino et al. (26) also orally immunized using live NPAP and heat-killed *B. abortus* 2308, while they orally challenged using *B. abortus* 2308. The results showed that *O. anthropi* induced a strong lgA anti-*Brucella* response in serum and feces. Oral vaccination with NPAP induced high protection levels but reduced ones in mice vaccinated with heat-killed *B. abortus* 2308.

## Nanoparticles

The oral administration with Omp31-loaded *N*-trimethyl chitosan nanoparticles in BALB/c mice induced a combination of Th1-Th17 responses, and a strong response of IFN-γ, IL-12, and IL-17. The mice challenged with *B. melitensis* 16M showed a significant level of protection in the spleen 4 weeks after vaccination (140).

## Outer Membrane Vesicles

It has been observed that Gram-negative bacteria can secrete membrane vesicles, which carry molecular components associated to the virulence of the bacterium they are derived from Dorneles et al. (107). Vesicles of Gram-negative bacteria derive from the outer membrane. The proteomic analysis of the outer membrane vesicles (OMVs) of *B. melitensis* 16M proved that they contain, among others, Omp16, Omp19, Omp25, Omp31, Cu/Zn SOD, IalB, InvB GroES, and bp26 (141, 164). OMVs are recognized by membrane and cytoplasmic receptors as NOD receptors (nucleotide binding oligomerization domain-like receptors) in eukaryotic cells and induce immune response. OMVs obtained from strain VTRM1 induced IL-12, TNF-α, and IFN-γ response significantly higher than that induced by strain 16M in DC derived from bone marrow. OMVs obtained from *B. melitensis* 16M were intraperitoneally applied as vaccine to BALB/c mice and induced a slightly reduced protection level against challenge with 16M, when compared with the challenge with *B. melitensis* Rev1 (2.64 vs. 1.9 logarithmic units). Interestingly, OMVs obtained from mutant rough *B. melitensis* VTRM1 conferred a much higher protection level (3.08 logarithmic units) (141). These results are promising since they suggest the possibility of obtaining an acellular vaccine to be applied in humans. However, its efficacy must be proven orally as an edible vaccine to induce local immunity, which must stop infection at the entry site.

## Concluding Remarks

The studies discussed in this review indicate current research on interactions between *Brucella* and its host mucosa. *Brucella* spp. is a pathogen that enters its host preferably through the mucosa of the gastrointestinal tract. Infection is eventually reported through the respiratory tract, either in the laboratory by inhalation of contaminated aerosols, or in the field by inhalation of dust particles including bacteria. Systemic immunity is well-known, since most experimental studies administer the bacteria intraperitoneally, where it establishes interactions with cellular populations different from those found in mucous membranes. As discussed, some studies suggest that in the intestine *Brucella* spp. interacts with dendritic cells, and that these represent a variety of populations with antagonistic functions. It has been observed that an important population of dendritic cells induces tolerogenic responses, so the role of dendritic cells with proinflammatory function should be studied.

In systemic infection the proinflammatory response is fundamental to establish the anti *Brucella* immunity, as it activates Th1 cells, which through the IFN-g stimulate the bactericidal activity of macrophages. In the mucosa a reaction must be subject to strong control as it can be deleterious. The role of cytotoxic T lymphocytes should be explored in more detail, as in systemic immunity studies they are essential to eliminate cells infected with *Brucella* spp. Preliminary results suggest their activation in the gut, but the role of Th17 cells in the gut as well as in the oral cavity and gut should be confirmed. It is possible that these cellular populations have a critical participation in these sites in view of the evidence suggesting that mucous membranes are reservoir sites for *Brucella* spp. which could be an important basis for the chronicity of *Brucella* infection. The study in some of these areas is growing exponentially and may translate into new vaccines and therapies. The new vaccines are involving the use of compounds derived from specific bacteria or bacteria as probiotics that protect mucosal surfaces and induce immunity.

New vaccines should increase protection against brucellosis, an intracellular bacterial infection that is difficult to treat. Much remains to be learned from the study of the interaction between *Brucella* and his host. The knowledge gained from these studies should also be translated into better therapeutic treatments to protect against this important chronic infectious disease.

## Author Contributions

RL-S contributed with the T-cell immunity and compilation of the paper's chapters. AS-A contributed with the chapter related to gut immunity. LD contributed with the chapter related to *Brucella* vaccines. SB-U contributed with the chapter related to respiratory tract immunity. MM-L contributed with the whole organization, the critical review and edition.

### Conflict of Interest Statement

The authors declare that the research was conducted in the absence of any commercial or financial relationships that could be construed as a potential conflict of interest.
